# Shady business: understanding the spatial ecology of exophilic *Anopheles* mosquitoes

**DOI:** 10.1186/s12936-018-2499-7

**Published:** 2018-10-05

**Authors:** Yared Debebe, Sharon R. Hill, Habte Tekie, Rickard Ignell, Richard J. Hopkins

**Affiliations:** 1 Department of Zoological Sciences, Addis Ababa University, PO. Box 1176, Addis Ababa, Ethiopia; 2 Unit of Chemical Ecology, Department of Plant Protection Biology, Swedish University of Agricultural Sciences, Alnarp, Sweden; 3 Natural Resources Institute, University of Greenwich, London, UK

**Keywords:** Exophilic, *Anopheles*, Landscape, Canopy, Land cover

## Abstract

**Background:**

Understanding the ecology of exophilic anophelines is a key step toward developing outdoor control strategies to complement existing indoor control tools against malaria vectors. This study was conducted to assess the movement pattern of exophilic *Anopheles* mosquitoes between blood meal sources and resting habitats, and the landscape factors dictating their resting habitat choice.

**Results:**

Resting clay pots were placed at 5 m, 25 m, 50 m, 75 m and 100 m away from isolated focal houses, radiating from them in four directions. The locations of the clay pots represent heterogeneous land cover types at a relatively fine spatial scale in the landscape. The effect of the landscape characters on the number of both female and male anophelines caught was modelled using zero-inflated negative binomial regression with a log link function. A total of 420 *Anopheles* mosquitoes (353 females and 67 males) belonging to three species; *Anopheles arabiensis*, *Anopheles pharoensis*, and *Anopheles tenebrosus* were caught in the resting clay pots, with *An. arabiensis* being the dominant species. Canopy cover, distance from the house, and land cover type were the significant landscape characters influencing the aggregation of resting mosquitoes. Both the count and binary models showed that canopy cover was the strongest predictor variable on the counts and the presence of *Anopheles* mosquitoes in the clay pots. Female *Anopheles* were most frequently found resting in the pots placed in banana plantations, and at sampling points that were at the greater distances (75 m and 100 m) from the focal house.

**Conclusions:**

This study showed that exophilic *Anopheles* mosquitoes tend to rest in shaded areas some distance away from human habitation. These findings are important when targeting mosquitoes outdoors, complementing the existing effort being made to control malaria vectors indoors.

**Electronic supplementary material:**

The online version of this article (10.1186/s12936-018-2499-7) contains supplementary material, which is available to authorized users.

## Background

Current interventions targeting indoor malaria vectors, particularly the use of long-lasting insecticidal nets (LLINs) and indoor residual sprays (IRS), have been a cornerstone of the recent significant decline in malaria morbidity and mortality [1]. As a result, malaria-related deaths have declined by more than half in sub-Saharan Africa between 2000 and 2015 [1, 2]. The sustainability of these interventions is, however, threatened due to increased vector resistance to available insecticides [3–5], and the change in mosquito biting behaviour to seeking blood meals outdoors [6–8], with some populations shifting the time of biting activity from late night to early evening [8–10]. These behavioural changes favour residual malaria transmission, presenting a major roadblock to further reduce malaria prevalence and enhance the sustainability of malaria vector control [11]. Whilst the current strategy of IRS and ITN control has made great strides against malaria, the global number of malaria cases has not declined in the past few years, but rather has increased by 5 million over the course of a single year in 2016, with no reduction in mortality evident for the first time in a decade [12]. Outdoor interventions directed against adult mosquitoes are lacking [13], and an increased understanding of the ecology and behaviour of exophilic malaria vectors is needed to improve the sustainability of existing control strategies. In addition, this may further act as a guide for the deployment of appropriate outdoor monitoring and control tools [14].

The sustainability of existing integrated vector management (IVM) tools should be actively maintained, and enhanced by the addition of novel interventions, particularly vector control strategies targeting adult anophelines outdoors [13, 15]. Early studies by Gillies [16, 17] revealed that endophilic *Anopheles gambiae* sensu lato (s.l.), the primary malaria vector at this time, predominantly rested indoors, but with a small proportion of mosquitoes found to be resting in shady zones at some distance from human habitation. In the interim, changes in the biting patterns of several mosquito species have arisen, whereby a far greater proportion of female *Anopheles* species are found to both feed and rest outdoors [6–10]. Additionally, the habitat has undergone considerable changes, populations of humans are denser, and the agricultural environment is more intensely farmed with greater use of irrigation [18]. In view of the known changes in mosquito feeding behaviour and the habitat, few recent studies describing the outdoor behaviour of mosquitoes have been conducted [19], which may be partly due to the large effort required to catch mosquitoes outdoors as opposed to indoors [20]. Existing knowledge builds extensively on the foundation of the work of Gillies [16] who studied the resting site selection of *An. gambiae* s.l. and *Anopheles funestus* in natural and artificial resting sites. More recent studies in *Anopheles* mosquitoes show that these mosquitoes choose outdoor resting micro-habitats based on several different environmental factors within the landscape at a fine spatial scale [21]. Moreover, a number of studies have associated landscape characters with the distribution or aggregation of exophilic mosquitoes [22–24]. These studies have indicated that different physical and biological components of the environment are important factors affecting mosquito ecology, with habitat type [22], land cover [23], shade [24], microclimate [21] and the availability of blood meal hosts [22] being positively associated with the adult distribution of exophilic mosquito species.

Outdoor monitoring and control tools can be used alone, or to augment other IVM strategies, to alleviate the malaria burden. It is, however, essential to fully understand the behaviour of exophilic populations to make the best use of both existing and novel tools. This study was conducted to explore the resting habitat selection behaviour of *Anopheles* mosquitoes outdoors and identify landscape characteristics associated with the resting sites which can later be used to optimize the positioning of traps in the landscape around human habitations.

## Methods

### Study area description

**Fig. 1:**
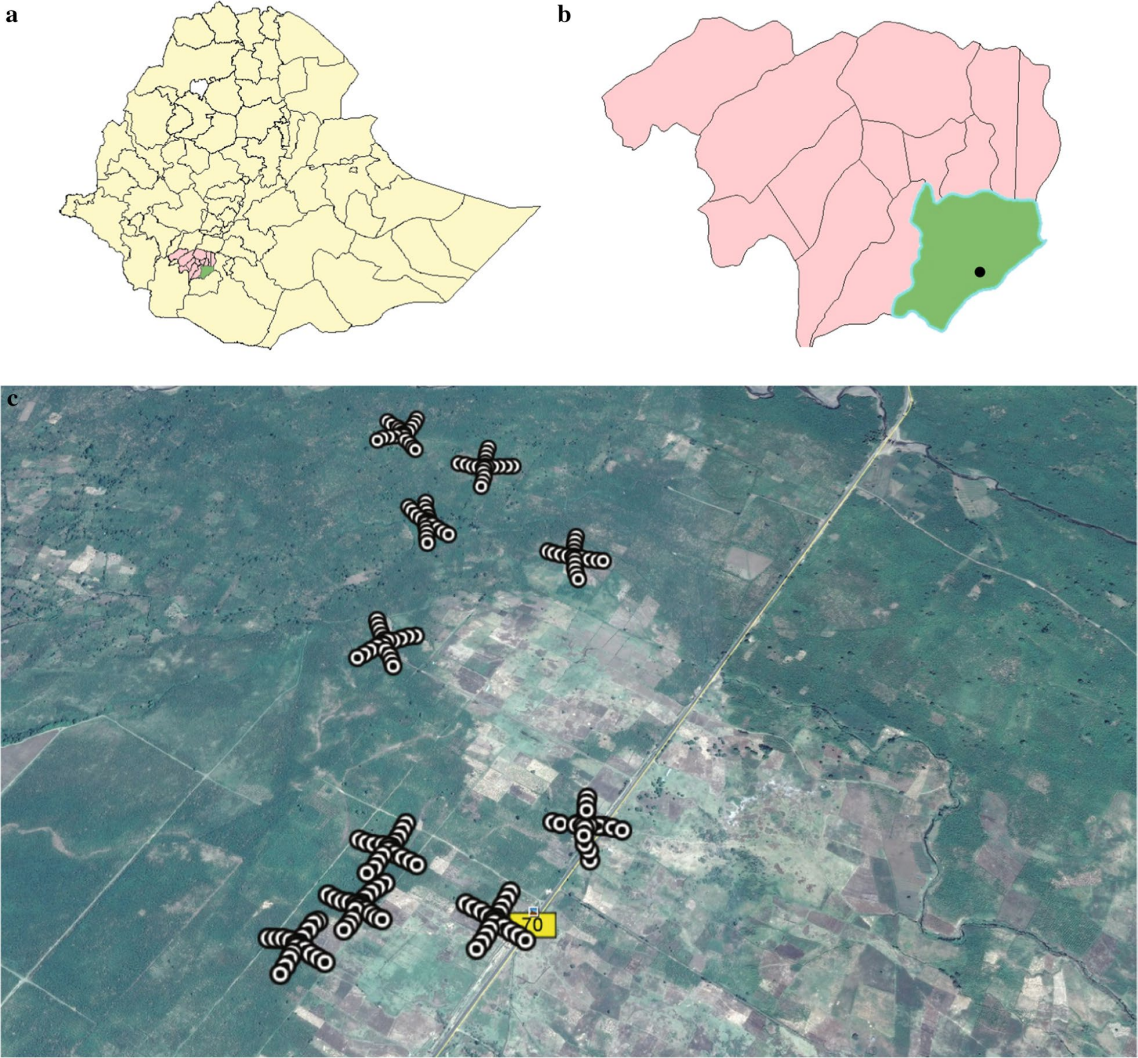
Maps showing **a** district map of Ethiopia indicating the Gamo-Gofa zone; **b** the Gamo-Gofa zone indicating Arba Minch Zuria district; and **c** the study area with the sampling points

The study was conducted in southern Ethiopia in Arba Minch Zuria district of the Gamo Gofa zone near a village called Sile (5°53′24′′N, 37°29′24′′E) (Additional file 1). The study site is 517 km south of Addis Ababa, the capital city of Ethiopia, and 17 km south of the city of Arba Minch, the capital of Gamo-Gofa zone (Fig. 1). The area is characterized by bimodal rainy seasons with a long rainy period between the months of April and June, and a short rainy season between September and October. This study was conducted between September 2016 and June 2017. The annual rainfall ranges from 900 to 1300 mm, and the average annual temperature is 25 to 36 °C. Banana is the main commercial crop in the area and covers approximately half of the landmass. Maize is cultivated predominantly for subsistence and makes up approximately 20% of the land used. The presence of abundant irrigation canals in the study area, and its proximity to Lake Chamo, creates suitable breeding sites for malaria vectors, making it one of the areas with the highest malaria transmission in the Gamo Gofa zone (based on personal communication with the district health officer). Livestock rearing, including both cattle and small ruminants, is a major activity in the area, and provides potential blood meal sources for mosquitoes.

### Study design and mosquito collection

**Fig. 2:**
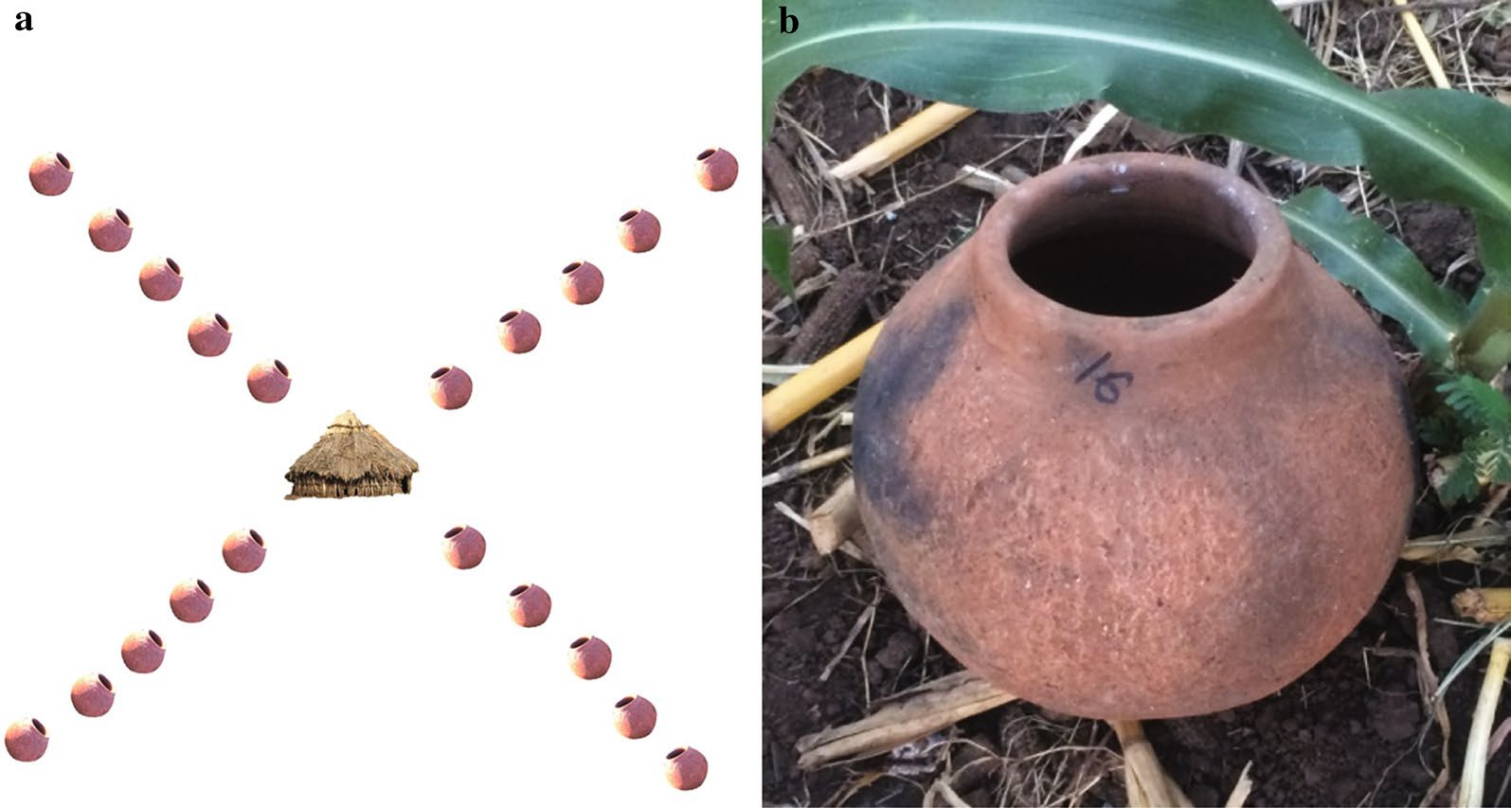
Schematic representation of the clay pot arrangement for collecting outdoor resting *Anopheles* mosquitoes (**a**) and a resting clay pot (**b**)

In order to identify the environmental factors affecting outdoor resting site selection by *Anopheles* mosquitoes, resting clay pots (Fig. 2a, b) were used to collect adult mosquitoes. The clay pots were spherical in shape and made to our specifications by local potters. The pots had an opening of approximately 15 cm, a depth of 40 cm and a capacity to hold ca. 10 l. A 2 cm hole was made at the bottom of the pots in order to avoid rain water accumulation and potential theft.

Ten isolated, inhabited houses, located a minimum of 200 m apart, were selected for the study. The selected houses had mud plastered walls with grass thatched roofs. Twenty clay pots were placed in a criss-cross pattern, with the house at the centre, and single pots being placed at 5 m, 25 m, 50 m, 75 m and 100 m away from the house in each of the four directions (Fig. 2). The hill side of the village was used to orient the position of the pots (Fig. 1).

### Environmental variables

Landscape characteristics were determined within a 10 m radius from each sampling points: (1) the distance of the sampling point from the nearest house with potential blood meal sources; (2) the number of potential breeding sites; (3) the land cover type and the percentage canopy cover; and (4) the relative percentage of ground (grasses and other herbs) and tall (shrubs and trees) vegetation. The geographical location of each sampling point and the houses were recorded using a handheld GPS instrument (Additional file 1).

### Mosquito sampling and identification

Sampling of mosquitoes was conducted in the morning between 06:00 and 09:00. During collection, a mosquito cage (BugDorm 32.5 cm × 32.5 cm × 32.5 cm) was placed over the opening of the clay pot, and by gently lifting and shaking the pot, as well as blowing air through the small opening at the bottom of the pot, the resting mosquitoes were encouraged into the cage. Then, the mosquitoes were aspirated from the cage, knocked down using ethyl acetate, and transported to the field laboratory.

The collected mosquitoes were counted and sorted according to species group and sex. Female mosquitoes were morphologically identified to species following Verrone [25] and Gillies and Coetzee [26], and subsequently categorized according to their abdominal status as unfed, blood fed, semi-gravid or gravid, following the categories defined by the World Health Organization [27]. Female *Anopheles* mosquitoes, provisionally identified as *An. gambiae* s.l., were individually preserved in 1.5 ml Eppendorf tubes containing silica gel and stored at ambient temperature for subsequent molecular identification to sibling species. Molecular identification of female *An. gambiae* s.l. was conducted using the species-specific polymerase chain reaction (PCR) technique described by Scott et al. [28].

### Data analysis

Data analysis was conducted using R statistical software version 3.4.1 [29] and JMP^®^ version 10.0.0. (SAS Institute Inc., Cary, NC, USA). As the response variable was an over-dispersed count data with unequal mean and variance, and due to the excess number of zero captures, a zero-inflated negative binomial regression with log-link function was used to model the effect of environmental factors on the number of outdoor resting *Anopheles* mosquitoes caught. Before conducting the regression analysis, a multiple correlation analysis was conducted to assess multicollinearity among the continuous predictor variables. Since canopy cover was positively correlated with the percentage of tall vegetation within a 10 m radius of the sampling points, the percentage of tall vegetation was removed from the subsequent model. A pairwise non-parametric Kruskal–Wallis was followed by Wilcoxon pairwise comparison post hoc test to compare the number of mosquitoes between the categories: land cover, shading, and distance from the focal house. A binomial logistic regression was conducted to predict the probability of catching at least a single *Anopheles* mosquito in the clay pots, followed by a backward selection of non-significant independent variables to model the count and binary outcomes.

## Results

### Mosquito abundance and physiological state

**Fig. 3:**
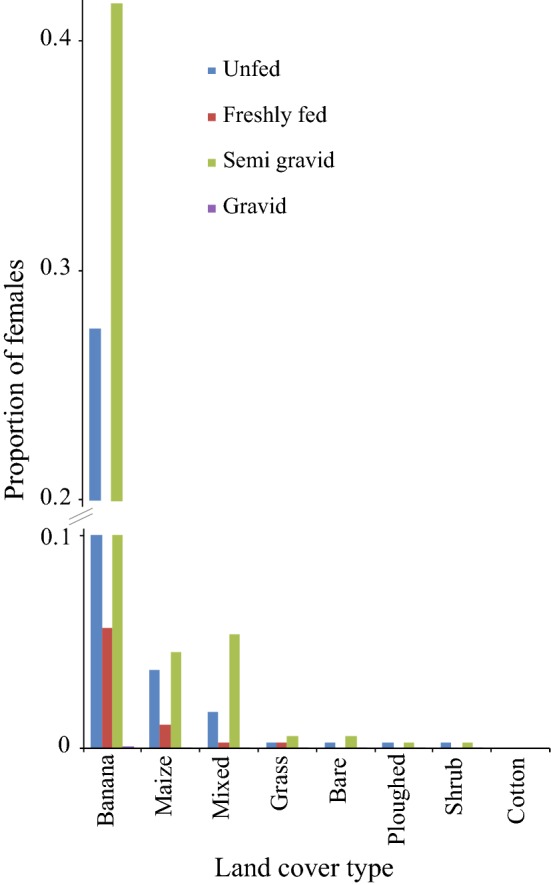
Proportion of different physiological states of *Anopheles* mosquitoes caught in clay pots distributed amongst different land cover types

Surveillance of resting *Anopheles* mosquitoes was conducted in a rural Ethiopian setting (Fig. 1) using clay pots as artificial resting sites (Fig. 2). A total of 420 *Anopheles* mosquitoes (353 females and 67 males) were caught in the clay pots. Three *Anopheles* species/species complexes were collected, of which *An. gambiae* s.l. was the most abundant species with 370 (88.1%) mosquitoes, followed by *Anopheles pharoensis* consisting of 49 individuals (11.67%) and *Anopheles tenebrosus* with a single individual (0.23%). Molecular identification of *An. gambiae* s.l. using PCR was conducted on 63 individuals (17%) identifying all mosquitoes as *Anopheles arabiensis*. The physiological state of female anophelines collected from each of the land cover types demonstrated that the highest proportions caught were semi-gravid, followed by unfed (Fig. 3).

### Effect of landscape elements on mosquitoes caught

The association between the number of *Anopheles* mosquitoes caught and the landscape characteristics, within a 10 m radius from each sampling point, was modelled using zero-inflated negative binomial regression (log-likelihood = − 264.8; df = 13; theta = 1.19) for females and (log-likelihood = − 110.8; df = 13; theta = 1.48) for males; (Additional file 2). Backward selection of non-significant independent variables indicated that percent canopy cover (P < 0.001) and distance of sampling points from the nearest dwelling (P < 0.01) significantly affected the number of female *Anopheles* mosquitoes caught in the resting clay pots, as indicated from count model coefficients in the model (Table 1). Both variables are the dominant characteristics of the banana-dominated land cover, where the highest *Anopheles* density was recorded. The result from the zero-inflation model also indicated that the odds of having an excess number of zeroes decreased with increasing percent canopy coverage and distance of sampling points from the focal house (Table 1). In contrast, none of the predictor variables from either the count or the zero-inflation models significantly affected the male *Anopheles* caught (Table 1).

**Table 1:** The effect of landscape characteristics within a 10 m radius of the sampling points on the number of *Anopheles* mosquitoes caught in resting clay pots, as shown by zero-inflated negative binomial regression, followed by backward selection of non-significant independent variables

Variables	Estimate	Std. error	z value	Pr(> |z|)
Females				
Count model coefficients (negbin with log link)				
(Intercept)	− 1.2435	0.6274	− 1.982	0.04747*
Distance to nearest dwelling (m)	0.0136	0.0052	2.599	0.0094**
Percent canopy cover	0.0238	0.0068	3.483	0.0005***
Zero-inflation model coefficients (binomial with logit link)				
(Intercept)	1.3602	0.6274	1.640	0.1010
Distance to nearest dwelling (m)	− 0.0016	0.0087	− 0.185	0.8530
Percent canopy cover	− 0.0279	0.0094	− 2.963	0.0030**
Males				
Count model coefficients (negbin with log link)				
(Intercept)	− 1.4289	1.2516	− 1.142	0.2536
Percent canopy cover	0.0215	0.0151	1.420	0.1555
Percent ground vegetation	− 0.0386	0.0231	− 1.672	0.0946
Zero-inflation model coefficients (binomial with logit link)				
(Intercept)	2.5915	1.5563	1.665	0.0959
Percent canopy cover	− 0.0432	0.0290	− 1.490	0.1361
Percent ground vegetation	0.0075	0.0503	0.149	0.8819

*negbin* negative binomial, *log link* logarithmic link, *logit link* logistic link

* P < 0.05, **P < 0.01, ***P < 0.001

### Effect of land cover, shade and distance from focal houses on mosquitoes caught

**Fig. 4:**
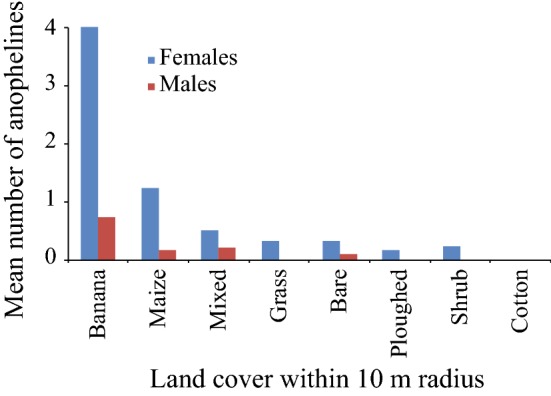
Mean number of *Anopheles* mosquitoes caught in the resting clay pots in different land cover types

The number of mosquitoes caught in the resting clay pots was compared among land cover types, as well as shading and distance categories from the focal houses. The analysis indicated that land cover type affected the number of both female (P < 0.0001) and male anophelines (P = 0.02) caught. Most of the mosquitoes were recorded in banana-dominated land cover for both sexes (Fig. 4). Shading also had a significant positive effect on the number of both females (P < 0.0001) and males (P < 0.0001). Clay pots placed in fully shaded areas caught a higher number of *Anopheles* mosquitoes than those positioned in partially shaded or non-shaded areas (Table 2). The number of *Anopheles* caught at a distance of 5 m, 25 m, 50 m, 75 m or 100 m radius from the focal houses was also compared revealing that the number of female *Anopheles* mosquitoes was higher at distances farther away from the focal house (P < 0.05). However, the distance of sampling points from the focal house had no significant effect on the number of male *Anopheles* caught (P > 0.05) (Table 2).

**Table 2:** The effect of categorical variables within a 10 m radius of the sampling points on the number of *Anopheles* mosquitoes caught in resting clay pots, as shown by Kruskal–Wallis test followed by Wilcoxon pair wise comparison method

Category	Number	Density of mosquitoes	
		Males	Females
Land cover			
Banana	69	0.72^a^	4.04^a^
Bare	12	0.08^b^	0.33^b^
Cotton	5	0.00^b^	0.00^b^
Grass	12	0.00^b^	0.33^b^
Maize	29	0.17^ab^	1.24^b^
Mixed	51	0.21^ab^	0.51^b^
Ploughed	13	0.00^b^	0.15^b^
Shrub	9	0.00^b^	0.22^b^
P-value		0.002	0.000
Shading			
Open	85	0.04^a^	0.14^a^
Partial	41	0.12^a^	1.29^b^
Shaded	74	0.80^b^	3.89^c^
P-value		0.000	0.000
Distance category			
Within 5 m	40	0.3	0.32^a^
Within 25 m	40	0.13	0.73^ac^
Within 50 m	40	0.25	2.15^bc^
Within 75 m	40	0.57	2.55^b^
Within 100 m	40	0.43	3.08^b^
P-value		0.429	0.0115

^abc^Values within each category in the same column, followed by the same letter are not significantly different (P > 0.05)

**Fig. 5:**
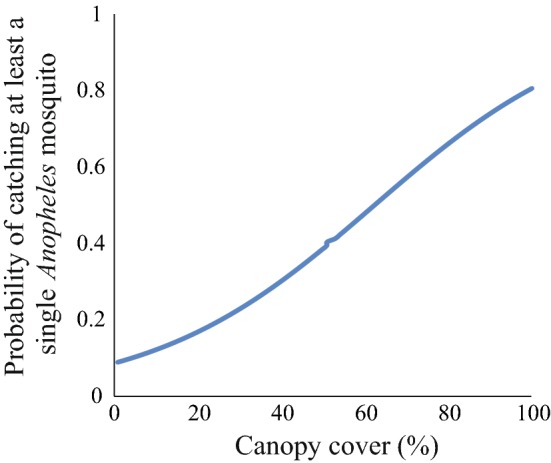
Estimated probability of catching at least one single *Anopheles* mosquito in relation to percent canopy coverage

The probability of catching at least a single *Anopheles* mosquito in the resting clay pots increased with an increasing percentage of canopy cover (P < 0.0001). The rest of the environmental factors had no significant effect on the probability of catching at least one *Anopheles* mosquito (P > 0.05). The model showing the effect of all predictor variables on the number of mosquito caught is indicated in Additional file 3, and Table 3 shows the model after removing the non-significant predictor variables. The estimated probability of catching at least one single anopheline in relation to canopy coverage is indicated in Fig. 5.

**Table 3:** The effect of percentage canopy cover within a 10 m radius of the sampling points on the presence or absence of *Anopheles* mosquitoes in resting clay pots, as shown by binary logistic regression

Variables	Estimate	Std. error	z value	Pr(> |z|)
(Intercept)	− 2.3495	0.3633	− 6.466	0.0011***
Percent canopy cover	0.0377	0.0059	6.389	0.00003***

## Discussion

This study found that the distance of the sampling points from the focal house, the percentage of canopy cover, as well as the land cover characteristics are important landscape predictor variables influencing the resting site selection of exophilic female *Anopheles* mosquitoes, particularly *An. arabiensis*. Similarly, canopy and land cover are important factors for male *Anopheles*. This study reveals that female *Anopheles* mosquitoes fly 50–100 m away from their blood feeding environment, in contrast to males, to rest in favoured habitats, primarily banana plantations, but also maize fields, which provide optimal shade cover for both males and females. This knowledge is an important step in understanding movement patterns of *Anopheles* mosquitoes and provides a foundation for further studies on the development of intervention strategies that can complement the IRS and ITNs.

Among the significant explanatory variables in our study, shade is the strongest driver of the distribution of exophilic female *Anopheles* mosquitoes in the landscape, in line with previous studies on other mosquito species [16, 30, 31]. The two *Anopheles* species in this study share a preference for shaded resting sites with other *Anopheles* species in different geographical locations throughout the tropical and subtropical regions of the world [32]. This preference for shaded areas has been linked to the avoidance of excess water loss, as dehydration negatively influences mosquito physiology, survival and fitness [33, 34].

Despite a lack of a statistically significant difference with other land cover types, the maize-dominated areas caught the second highest number of *Anopheles*. It is noteworthy that maize cultivations have been shown, in this and other regions of eastern Africa, to harbour a large number of resting mosquitoes (*personal observation*). This is likely due to the fact that maize provides relatively high levels of shade, up to 2 m in height, in comparison with the other land cover classes, where mosquito abundance was found to be low or non-existent. Moreover, it has been shown that there is a direct link between the breeding sites and malaria prevalence during maize and other cereal crop irrigated cultivation [35–38]. The main driver for this is the maize pollen, which provide an important food source for mosquito larvae, increasing the chance of survivorship and higher pupation rate [39]. The adults that emerge from well-nourished larvae are larger in size, less susceptible to chemical insecticides, show increased biting frequency, and have longer blood meal duration and longevity; all of these biological traits are positively contributing to the vectorial capacity of the adult mosquitoes [35, 39–41].

The distance of the sampling points from the nearest house had a positive effect on the number of female *Anopheles* mosquitoes caught, with catches being higher further away from the house. One likely explanation of this is that sampling clay pots placed near the houses had fewer mosquitoes due to the recurrent disturbance by human and livestock activities. Furthermore, canopy cover, as the strongest predictor variable, is associated with dense banana cultivation, which is located further away from the houses. Thus, female mosquitoes may be motivated to fly a longer distance to reach a shaded refuge. This is in line with previous research, which studied the spatial movement pattern of mosquitoes from the edge of a forest into the interior [42]. Mendez et al. [42] demonstrated that mosquitoes aggregated 100 m and 200 m from the forest edge, leaving the high disturbance, low shade area. One of the pioneer works in understanding the outdoor resting behaviour of *Anopheles* mosquitoes was conducted by Gillies [16]. The author studied the outdoor resting behaviour of *An. gambiae* s.l. by using artificially constructed resting boxes placed at different distances from residential houses. The results indicated that resting boxes placed at distant positions caught a higher number of *An. gambiae* s.l. than resting boxes placed near the houses. However, most of the resting *An. gambiae* sensu stricto. mosquitoes were caught indoors. The findings of the present study are in partial agreement with the work of Gillies [16], finding that outdoor resting *An. arabiensis* also prefer heavily shaded resting sites providing optimal microclimate for blood meal digestion.

## Conclusion

Previous studies aimed at modelling the effect of landscape characteristics on the distribution of mosquitoes have used a relatively large spatial scale of up to 1000 m to analyse the position of mosquitoes in the landscape [43, 44]. Findings presented in this study show that fine-scale spatial heterogeneity of landscape structures affects the distribution or aggregation of *Anopheles* mosquitoes, in line with studies on *Culex pipiens estuans* [45]. Here, the landscape characters are shown to be important drivers of movement patterns and resting site selection of exophilic mosquitoes. In this era of the uncertain sustainability of two major vector control strategies, IRS and ITNs, the search for novel vector control options particularly targeting outdoor populations is of great importance. Knowledge of the mosquito ecology is critical for further studies intended to develop novel monitoring and control tools that work for outdoor feeding and resting *Anopheles* populations.

## Additional files

**Additional file 1.** Elevation and geographical location of each sampling point.

**Additional file 2.** Results obtained from zero-inflated negative binomial regression on the association between the number of *Anopheles* mosquitoes caught and landscape characteristics within a 10 m radius of the sampling points.

**Additional file 3.** Results obtained from binary logistic regression on the association between the presence or absence of *Anopheles* mosquitoes and landscape characteristics within a 10 m radius of the sampling points.

## Abbreviations

df: degrees of freedom
IRS: indoor residual sprays
ITN: insecticide treated nets
IVM: integrated vector management
LLINs: long lasting insecticidal nets
PCR: polymerase chain reaction
