# Supplementary material for: Shady business: understanding the spatial ecology of exophilic Anopheles mosquitoes
Source: Malar J. 2018 Oct 5;17:351. doi: 10.1186/s12936-018-2499-7 (PMC6173902; doi:10.1186/s12936-018-2499-7)
Supplement: Supplementary file 1 — Additional file 1. Elevation and geographical location of each sampling point. [file 12936_2018_2499_MOESM1_ESM.pdf]

Additional file 1. Elevation and geographical location of each sampling point.

Sampling point ID	Location °N	Location °E	Altitude (m)
HH1-1	5.886	37.49152778	1117
HH1-2	5.886194444	37.49152778	1120
HH1-3	5.886416667	37.49155556	1119
HH1-4	5.886638889	37.49155556	1118
HH1-5	5.886833333	37.49158333	1116
HH1-6	5.885916667	37.49144444	1116
HH1-7	5.885944444	37.49125	1116
HH1-8	5.885972222	37.49130556	1112
HH1-9	5.885972222	37.49080556	1115
HH1-10	5.886027778	37.49058333	1119
HH1-11	5.885833333	37.4915	1117
HH1-12	5.885611111	37.49147222	1118
HH1-13	5.885333333	37.4915	1117
HH1-14	5.885138889	37.49161111	1117
HH1-15	5.884833333	37.49163889	1115
HH1-16	5.885916667	37.49161111	1118
HH1-17	5.885916667	37.49188889	1117
HH1-18	5.885916667	37.49205556	1117
HH1-19	5.885861111	37.49227778	1117
HH1-20	5.885777778	37.49255556	1117
HH2-1	5.883027778	37.489	1116
HH2-2	5.883138889	37.48886111	1117
HH2-3	5.883277778	37.48866667	1119
HH2-4	5.883388889	37.48844444	1117
HH2-5	5.8835	37.48825	1118
HH2-6	5.883083333	37.48908333	1119
HH2-7	5.88325	37.48916667	1119
HH2-8	5.883444444	37.48925	1119
HH2-9	5.883666667	37.48936111	1118
HH2-10	5.883861111	37.48944444	1119
HH2-11	5.882972222	37.48911111	1118
HH2-12	5.882833333	37.48930556	1118
HH2-13	5.882694444	37.4895	1116
HH2-14	5.882583333	37.48969444	1115
HH2-15	5.882444444	37.48988889	1116
HH2-16	5.882944444	37.48902778	1117
HH2-17	5.882777778	37.48891667	1117
HH2-18	5.882583333	37.48883333	1115

Sampling point ID	Location °N	Location °E	Altitude (m)
HH2-19	5.882388889	37.48872222	1115
HH2-20	5.882194444	37.48858333	1117
HH4-1	5.8835	37.48538889	1120
HH4-2	5.883666667	37.4855	1119
HH4-3	5.883833333	37.48563889	1122
HH4-4	5.883972222	37.48580556	1122
HH4-5	5.884111111	37.486	1122
HH4-6	5.883472222	37.48525	1119
HH4-7	5.883527778	37.48505556	1121
HH4-8	5.883555556	37.48486111	1121
HH4-9	5.883638889	37.48466667	1121
HH4-10	5.88375	37.48447222	1122
HH4-11	5.883388889	37.48530556	1119
HH4-12	5.883305556	37.48525	1121
HH4-13	5.883027778	37.48519444	1122
HH4-14	5.882861111	37.48511111	1120
HH4-15	5.882722222	37.485	1119
HH4-16	5.883416667	37.48538889	1121
HH4-17	5.883305556	37.48555556	1119
HH4-18	5.883194444	37.48569444	1119
HH4-19	5.883083333	37.48583333	1117
HH4-20	5.883027778	37.486	1117
HH7-1	5.885222222	37.48608333	1127
HH7-2	5.885361111	37.48616667	1127
HH7-3	5.885555556	37.48627778	1127
HH7-4	5.885777778	37.48636111	1123
HH7-5	5.885972222	37.48644444	1121
HH7-6	5.885222222	37.486	1116
HH7-7	5.885277778	37.48583333	1118
HH7-8	5.885305556	37.48558333	1120
HH7-9	5.885361111	37.48536111	1119
HH7-10	5.885444444	37.48516667	1121
HH7-11	5.885111111	37.486	1119
HH7-12	5.884972222	37.48586111	1122
HH7-13	5.884777778	37.48575	1124
HH7-14	5.884583333	37.48566667	1126
HH7-15	5.884416667	37.48547222	1125
HH7-16	5.885111111	37.48608333	1114
HH7-17	5.885	37.48622222	1115
HH7-18	5.884888889	37.48641667	1116

Sampling point ID	Location °N	Location °E	Altitude (m)
HH7-19	5.884777778	37.48663889	1117
HH7-20	5.884666667	37.48683333	1122
HH8-1	5.882333333	37.48383333	1119
HH8-2	5.8825	37.48391667	1122
HH8-3	5.882722222	37.48405556	1122
HH8-4	5.882916667	37.48416667	1122
HH8-5	5.874777778	37.48427778	1123
HH8-6	5.882305556	37.48375	1122
HH8-7	5.882416667	37.48358333	1121
HH8-8	5.882472222	37.48338889	1122
HH8-9	5.882583333	37.49983333	1120
HH8-10	5.882666667	37.49966667	1122
HH8-11	5.882222222	37.48377778	1122
HH8-12	5.882055556	37.48369444	1121
HH8-13	5.881916667	37.48363889	1122
HH8-14	5.881694444	37.48355556	1121
HH8-15	5.881472222	37.48344444	1122
HH8-16	5.88225	37.48386111	1118
HH8-17	5.882166667	37.48402778	1119
HH8-18	5.882027778	37.48419444	1117
HH8-19	5.881916667	37.48438889	1116
HH8-20	5.881805556	37.48458333	1116
HH3-1	5.89228	37.48524	1121
HH3-2	5.89217	37.48511	1121
HH3-3	5.89203	37.48492	1119
HH3-4	5.89188	37.48474	1120
HH3-5	5.89174	37.48454	1120
HH3-6	5.89222	37.48532	1120
HH3-7	5.89202	37.48539	1120
HH3-8	5.89182	37.48553	1120
HH3-9	5.89162	37.4856	1119
HH3-10	5.89142	37.48567	1118
HH3-11	5.89239	37.48542	1122
HH3-12	5.89239	37.48561	1121
HH3-13	5.89248	37.48584	1118
HH3-14	5.89256	37.48605	1121
HH3-15	5.89259	37.48628	1119
HH3-16	5.89243	37.48532	1121
HH3-17	5.89259	37.48523	1119
HH3-18	5.89279	37.48515	1120

Sampling point ID	Location °N	Location °E	Altitude (m)
HH3-19	5.89299	37.48508	1120
HH3-20	5.89320	37.48503	1123
HH5-1	5.89721	37.48631	1117
HH5-2	5.89702	37.48633	1117
HH5-3	5.89683	37.48637	1119
HH5-4	5.89661	37.48637	1116
HH5-5	5.89639	37.48641	1115
HH5-6	5.89730	37.48631	1117
HH5-7	5.89744	37.48614	1114
HH5-8	5.89759	37.48597	1116
HH5-9	5.89780	37.48581	1115
HH5-10	5.89796	37.48569	1119
HH5-11	5.89724	37.48643	1116
HH5-12	5.89717	37.48653	1114
HH5-13	5.89701	37.48672	1117
HH5-14	5.89687	37.4869	1114
HH5-15	5.89675	37.48708	1115
HH5-16	5.89735	37.48637	1115
HH5-17	5.89750	37.48637	1119
HH5-18	5.89773	37.48631	1115
HH5-19	5.89793	37.48624	1116
HH5-20	5.89817	37.48625	1120
HH6-1	5.89979	37.48825	1120
HH6-2	5.89981	37.48846	1118
HH6-3	5.89983	37.48868	1118
HH6-4	5.89983	37.48893	1117
HH6-5	5.89984	37.48914	1117
HH6-6	5.89984	37.48818	1117
HH6-7	5.90000	37.48818	1117
HH6-8	5.90023	37.48819	1117
HH6-9	5.90046	37.48821	1118
HH6-10	5.90069	37.48826	1116
HH6-11	5.89979	37.48812	1119
HH6-12	5.89980	37.48792	1119
HH6-13	5.89981	37.48772	1119
HH6-14	5.89990	37.48751	1118
HH6-15	5.89994	37.48727	1117
HH6-16	5.89972	37.48816	1117
HH6-17	5.89955	37.48813	1121
HH6-18	5.89933	37.48817	1117

**Sampling point ID**	**Location °N**	**Location °E**	**Altitude (m)**
HH6-19	5.8991	37.48812	1120
HH6-20	5.89889	37.48808	1120
HH9-1	5.89586	37.49122	1112
HH9-2	5.89604	37.49122	1115
HH9-3	5.89624	37.49123	1116
HH9-4	5.89647	37.49125	1114
HH9-5	5.89668	37.49129	1113
HH9-6	5.89582	37.49119	1113
HH9-7	5.89587	37.491	1114
HH9-8	5.89592	37.49077	1114
HH9-9	5.89599	37.49055	1114
HH9-10	5.89606	37.49037	1115
HH9-11	5.89573	37.49124	1115
HH9-12	5.89554	37.49125	1114
HH9-13	5.89531	37.49125	1114
HH9-14	5.89509	37.49129	1116
HH9-15	5.89489	37.49135	1114
HH9-16	5.8958	37.49131	1117
HH9-17	5.89578	37.49147	1115
HH9-18	5.89572	37.49171	1117
HH9-19	5.89568	37.49195	1115
HH9-20	5.89566	37.49216	1116
HH10-1	5.90134	37.4853	1122
HH10-2	5.90147	37.4844	1120
HH10-3	5.90168	37.48554	1121
HH10-4	5.90188	37.48563	1117
HH10-5	5.9021	37.48569	1119
HH10-6	5.90128	37.48518	1119
HH10-7	5.90124	37.48501	1119
HH10-8	5.9012	37.48478	1117
HH10-9	5.90115	37.48456	1117
HH10-10	5.90103	37.48439	1116
HH10-11	5.90125	37.4853	1117
HH10-12	5.90106	37.48537	1117
HH10-13	5.90087	37.48553	1119
HH10-14	5.90069	37.48565	1118
HH10-15	5.90059	37.48574	1121
HH10-16	5.90138	37.48524	1116
HH10-17	5.90153	37.48514	1117
HH10-18	5.90166	37.48505	1115

Sampling point ID	Location °N	Location °E	Altitude (m)
HH10-19	5.90181	37.48483	1115
HH10-20	5.90197	37.48467	1117
