# Supplementary material for: Shady business: understanding the spatial ecology of exophilic Anopheles mosquitoes
Source: Malar J. 2018 Oct 5;17:351. doi: 10.1186/s12936-018-2499-7 (PMC6173902; doi:10.1186/s12936-018-2499-7)
Supplement: Supplementary file 2 — Additional file 2. Results obtained from zero-inflated negative binomial regression on the association between the number of Anopheles mosquitoes caught and landscape characteristics within a 10 m radius of the sampling points. [file 12936_2018_2499_MOESM2_ESM.pdf]

Additional file 2. Results obtained from zero-inflated negative binomial regression on the association between the number of *Anopheles* mosquitoes caught and landscape characteristics within 10 m radius of the sampling points.

**Variables**	**Females**		**Males**	
	**Estimate**	**Pr(>|z|)**	**Estimate**	**Pr(>|z|)**
**Count model coefficients (negbin with log link)**				
(Intercept)	-1.3478	0.0326*	-1.3673	0.2419
Distance to nearest dwelling (m)	0.0132	0.0086**	0.0053	0.4724
Breeding sites (present)	-0.2112	0.8520	-1.3583	0.5030
Number of breeding sites	0.4284	0.6704	0.6199	0.6883
Percent canopy cover	0.0232	0.0004***	0.0199	0.2034
Percent ground vegetation	0.0110	0.2401	-0.0379	0.0814
**Zero-inflation model coefficients (binomial with logit link)**				
(Intercept)	1.5594	0.0685	2.1742	0.144
Distance to nearest dwelling (m)	-0.0030	0.7128	0.0171	0.326
Breeding sites (present)	-0.7048	0.7523	3.1967	0.549
Number of breeding sites	0.0956	0.9616	-3.3128	0.487
Percent canopy cover	-0.0283	0.0014**	-0.0444	0.098
Percent ground vegetation	0.0096	0.4803	0.0021	0.960

*p < 0.05, **p < 0.01, ***p <0.001
