# Supplementary material for: Shady business: understanding the spatial ecology of exophilic Anopheles mosquitoes
Source: Malar J. 2018 Oct 5;17:351. doi: 10.1186/s12936-018-2499-7 (PMC6173902; doi:10.1186/s12936-018-2499-7)
Supplement: Supplementary file 3 — Additional file 3. Results obtained from binary logistic regression on the association between the presence or absence of Anopheles mosquitoes and landscape characteristics within a 10 m radius of the sampling points. [file 12936_2018_2499_MOESM3_ESM.pdf]

Additional file 3. Results obtained from binary logistic regression on the association between the presence or absence of *Anopheles* mosquitoes and landscape characteristics within 10 m radius of the sampling points.

**Variables**	**Estimate**	**Std. Error**	**z value**	**Pr(>|z|)**
(Intercept)	-2.6140	1.139	-2.295	0.0217 *
Distance to nearest dwelling (m)	0.0003	0.006	0.068	0.9456
Breeding sites (present)	-0.0732	2.034	-0.036	0.9713
Number of breeding sites	0.4235	1.907	0.222	0.8242
Percent canopy cover	0.0321	0.008	4.154	0.0000 ***
Land cover (banana)	1.0090	1.185	0.851	0.3945
Land cover (bare)	0.7281	1.295	0.562	0.5740
Land cover (grass)	1.2520	1.562	0.802	0.4226
Land cover (maize)	0.2452	1.195	0.205	0.8375
Land cover (mixed)	0.4082	1.165	0.350	0.7260
Land cover (ploughed)	0.4134	1.342	0.308	0.7580
Percent ground vegetation	-0.0136	0.014	-0.970	0.3318
